# Outcomes associated with nurse practitioners in collaborative practice with general practitioners in rural settings in Canada: a mixed methods study

**DOI:** 10.1186/1478-4491-12-69

**Published:** 2014-12-11

**Authors:** Alison Roots, Marjorie MacDonald

**Affiliations:** School of Health and Human Sciences, Southern Cross University, PO Box 157, Lismore, NSW 2480 Australia; School of Nursing, University of Victoria, HSD A402, STN CSC, PO Box 1700, Victoria, BC V8W 2Y2 Canada

**Keywords:** Case study, Outcomes, Primary care nurse practitioner, Rural practice

## Abstract

**Background:**

The formalized nurse practitioner (NP) role in British Columbia is relatively new with most roles implemented in primary care. The majority of primary care is delivered by physicians using the fee-for-service model. There is a shortage of general practitioners associated with the difficulties of recruitment and retention, particularly in rural and remote locations. The uptake of the primary care NP role has been slow due to challenges in understanding the extent of its contributions. This study aims to identify the outcomes associated with the NP role in collaborative primary care practice.

**Methods:**

Three case studies where NPs were embedded into rural fee-for-service practices were undertaken to determine the outcomes at the practitioner, practice, community, and health services levels. Interviews, documents, and before and after data, were analyzed to identify changes in practise, access, and acute care service utilization.

**Results:**

The results showed that NPs affected how care was delivered, particularly through the additional time afforded each patient visit, development of a team approach with interprofessional collaboration, and a change in style of practise from solo to group practise, which resulted in improved physician job satisfaction. Patient access to the practice improved with increased availability of appointments and practice staff experienced improved workplace relationships and satisfaction. At the community level, access to primary care improved for harder-to-serve populations and new linkages developed between the practice and their community. Acute care services experienced a statistically significant decrease in emergency use and admissions to hospital (*P* = 0.000). The presence of the NP improved their physician colleagues’ desire to remain in their current work environment.

**Conclusions:**

This study identified the diversity of needs that can be addressed by the NP role. Namely, the importance of time to enhance patient care and its associated benefits, especially in the fee-for-service model; the value of the NP’s role in the community; the acceptance of the clinical competence of NPs by their physician colleagues; the outcomes generated at the practice level in terms of organizational effectiveness and service provision; and substantiated the impact of the role in improving primary care access and reducing acute care utilization.

## Background

Over the past 40 years, the nurse practitioner (NP) role has been introduced into the delivery of primary care services in North America, Europe, and Australasia [1, 2]. A proliferation of studies, including more than three dozen randomized control trials, have demonstrated that NPs can deliver a wide variety of effective primary care services [3], confirmed that they are safe health care providers [4–6], identified the significant contributions they make to improved health outcomes for patients [7–11], and established that there is a high level of patient satisfaction with the care provided [12–15]. Despite all this research, few studies have examined the impact of NP role implementation beyond the level of these individual patient and family outcomes [16, 17]. There is limited information available that describes and explains what changes can occur at the other levels of the organization or health care system as a result of introducing the NP role into primary care practices. Little is known about what outcomes result from these changes and what effect they have on general practitioners (GP), primary care practice, the community, and the utilization of the local acute care health system by the patients of the primary care practice [18, 19].

The introduction and sustainability of the NP role in primary care has been difficult in some countries [20, 21]. This has been attributed, in part, to the challenges of identifying and understanding the contributions the NP role makes in the delivery of primary care services and the larger health care system [22]. To make the contributions of the NP role more visible, the specific changes that occur as a result of implementing this role need to be identified and the associated outcomes recognized [23]; without this, it is difficult to demonstrate the value the NP role adds to the delivery of primary care services and the larger health care system [22, 24, 25].

In many countries, the introduction of the NP role has been closely tied to the availability of GPs to meet the public’s need for primary care services [21, 26, 27]. Primary care delivery systems have been under significant and increasing pressure for several decades [28–30]. Reform strategies for the overall healthcare system have emphasized the need for increased access to primary care, stressed a shift from a treatment focus to increased emphasis on health promotion, disease prevention, and community-based care, and have highlighted quality-related issues involving co-ordination, integration, and multidisciplinary care [29, 31]. This reform agenda has fuelled, and in some cases revived, interest in the NP role as part of the primary care system [32, 33].

Improving access to primary care is more effective in improving health outcomes for a population than relying on secondary and tertiary services [34]. However, recruitment of new physicians to primary care has been an on-going concern with the annual intakes into post-graduate programs being insufficient to maintain the necessary supply of GPs [35, 36]. Governments of many countries have identified difficulties in accessibility to primary care due to workforce shortages [30, 37, 38]. Declining interest in primary care medicine has been attributed to many factors including workload, lifestyle, and job satisfaction [39–41]. Improvements in the practice environment, more reasonable hours of work, and the presence of professional colleagues and support, have the most influence on improving recruitment and retention, particularly in rural settings [42].

Primary care services have impacts at multiple levels of the healthcare system, including the acute care sector, community services, the internal practice environment, and individual practitioner [16, 18, 32]. These impacts are further influenced by the interdependent relationships that exist among these levels [43]. The addition of non-physician clinicians into the primary care environment can affect these impacts at all levels [17, 44–46]; however, little is known about how or to what extent the NP role influences these multi-level impacts.

It is now well established that the quality of health care improves when the model of service delivery is based on a team approach involving collaboration between health care professionals [47–49]. Evidence has shown that collaborative practice between different health professionals, including NPs, pharmacists, physician assistants, and physicians, can, in some settings, produce positive results such as improvements in patient access, quality of services delivered, professional job satisfaction, and workplace productivity [33, 50–54]. A Canadian decision support synthesis on advanced practice nursing (NP and clinical nurse specialist roles), which included a scoping review of literature and key informant interviews, found that one of the most frequent and consistently identified challenges in NP role implementation was the nature of the working relationship between NPs and physicians [55]. If the working relationship between them was good, it became a key facilitator for role implementation and integration, if not, it became a significant barrier [56]. The decision support synthesis identified that while some evidence was beginning to accumulate about the benefits of this collaborative relationship in primary care settings, further research is needed to better understand the important contributions made by the addition of the NP role [55].

The primary health care reform agenda that has supported the introduction, or re-introduction, of the NP role in primary care has been associated, in part, with the need for more efficient use of resources, in terms of cost, in the provision of health care services [31, 57]. The NP role has been described in the literature as demonstrating cost-effectiveness for nearly three decades [58, 59], although this cost-effectiveness is more often associated with NP roles involving hospitalized, long term care, and transitioning patients [60–62]. Cost-effectiveness of the NP role in primary care settings is less well studied, with the majority of research being associated with the physician substitution model of the primary care NP role [59, 63, 64]. Studies have identified that the greatest cost-effective outcomes can be obtained by focusing on the most effective model of care rather than the type of practitioner. The most effective model appears to be care provided through collaboration between NPs and other health professionals [59, 65, 66], and care that has an increased emphasis on health promotion and chronic disease management that results in the reduction of costly hospitalizations [47].

In a number of countries, primary care in the community is largely provided by GPs using a fee-for-service (FFS) method of remuneration [37, 67]. The mechanism for remunerating the NP role in many countries is based on a salaried approach [16–18, 21]. The majority of studied NP roles in primary care are in community clinics which do not utilize the FFS method of remuneration [16, 17, 21]; as a result, little is understood and more research is needed to identify the type and extent of contributions the NP role can make in the FFS context, the merits and limitations of this model, and what outcomes have been achieved [68].

This evidence suggested that the addition of NPs working in a collaborative practice relationship with GPs in community-based primary care practices has significant potential to create positive outcomes across multiple levels of the health care system.

## Methods

### Aim

In this paper, we report on the findings of a Canadian study to identify the impact of NP role implementation at the practitioner, practice, community, and local acute care health services levels when a NP worked in collaborative practice with GPs in rural FFS primary care practices.

NPs across the world have different levels of education and scopes of practice [21]. In British Columbia, Canada, where this study took place, the NPs were prepared at the master’s level and had the authority to work independently and to treat patients with common acute and chronic conditions in general primary care settings. The NPs’ scope of practice allows them to independently diagnose, order diagnostic tests, prescribe medications and treat, or consult and referral to a physician if needed [69, 70].

### Design

The study used a case study design based on an ecological framework [71, 72]. A case study involves constructing detailed and intensive knowledge about a single case or a small number of related cases. A case is a “*phenomenon of some sort occurring within a bounded context*” ([73], p. 25) and represents the unit of analysis in this type of design. Multiple types and sources of data are typically used to explore, describe, and explain the phenomenon of interest within each case [74–76]. Multiple cases are used, when possible, to add confidence to the findings. According to Yin [74], this reflects replication logic and adds strength to the conclusions. The findings of each case are compared to the findings of the other cases using cross-case comparison techniques and data displays (e.g., tables, causal network maps) as described by Miles and Huberman [73].

An ecological framework was used to represent the nested relationships or levels of analysis within the cases. At the core of the ecological framework is the NP role, surrounded by nested concentric circles to represent the practitioner, practice, community, and health care system levels. Drawing from the literature, factors influencing or resulting from NP role implementation were identified and grouped within the appropriate level and used to theoretically sensitize data collection. At the stage of analysis, impacts of NP role implementation were identified and grouped according to their ecological level, and the relationships among NP actions and the impacts at each level were traced in the data. Theoretically, an ecological perspective suggests that factors in each level influence factors or components at the levels above and below it in reciprocal fashion. The analysis of the relationships in the ecological framework are not the focus of this paper but will be provided in a subsequent publication. Thus, an ecological framework reflects the multi-layered relationships and influences that were expected to exist between the NP role and the other practitioners, the practice organization, the community, and the acute care health system that interacted with the community-based primary care practice.

### Case selection

The study was conducted in a rural area in one of the five geographically-based health authorities (government organizations responsible for health service delivery) in the province. The case study sites were primary care practices in which a NP had been ‘embedded’ to work in a collaborative practice model with the FFS GPs. These NPs were salaried employees of the health authority, had been educated as family primary care providers at the master’s level, and were placed in these practices to strengthen the provision of primary care services in the community.

Across the three cases, 25 individuals participated representing NPs, GPs, other practice staff, other community-based health care providers, and health authority representatives. Descriptions of the participants are summarized in Table 1. Ethical approvals were obtained from the research ethics boards of both the health authority and the university and all participation in the study was voluntary. All archival records provided by the practice or health authority had any identifiable information removed before they were provided to the researcher. One restriction imposed by the health authority ethics review committee was that we could not reveal which type of health care provider made particular statements because of the concern that in a small regional health authority with very few NPs in practice that the study sites, and thus the individual participants, might be identifiable. This does make interpretation of the findings a bit more difficult for the reader, and represents one limitation of the study.

**Table 1:** **Participants per case**

Participant	Case 1	Case 2	Case 3
General practitioners	3	4	1
Nurse practitioner	1	1	1
Other practice staff (RNs/medical office assistants)	2	3	2
Community-based health care providers	2	3	2
Health authority representative	1	1	1
**Total participants**	**9**	**12**	**7**

### Data collection

The qualitative data were collected through interviews, direct observation, field notes, archival records, and documents. Semi-structured key informant interviews were undertaken with all participants to identify the components of the NP role and the positive and negative changes that had occurred since the introduction of the role in each setting. Direct observations included the physical arrangements of the practices, interactions between staff, and informal meetings; these were recorded as field notes. Documents were collected from both the government and the health authority relating to the implementation of the NP role, as well as newspaper, TV and radio clips, conference presentations, and letters.

The quantitative data comprised practice and health authority service records. Before and after records were obtained from both the practices and the health authority to identify changes in patient access to and the volume of patients served by the practice, and utilization of acute care health services (emergency services and hospital admissions from emergency) by the patients of these practices. The ‘before’ period was a 7-month period commencing between 7 and 11 months before the introduction of the NP to the practice. The NPs were introduced in either 2007 or 2008. The ‘after’ period was the same 7 months of 2011 to control for any seasonal variations that might impact on the utilization of these health services. Across the three cases, 11,524 occasions of acute care service utilization were included in the data.

### Data analysis

All data were analyzed using a parallel mixed data analysis strategy [77]. This strategy involved two separate processes; first, the qualitative and quantitative components were analysed separately but in parallel, using appropriate thematic analysis and descriptive/inferential statistics, respectively, then the findings of the two components were integrated to form meta-inferences or conclusions. This type of analysis is appropriate within a case study design in which an understanding of the case as a whole needs to be achieved. This process allowed the two databases to form a complete picture of the enacted role of the NP, its context, and the co-dependent patterns of the relationships as well as the changes that occurred at the different levels as a result of the NP role implementation at the study sites [78, 79]. This analysis was first conducted at the within-case level to explore and describe the findings for each case, and then a cross-case analysis was undertaken to identify similarities and differences among the cases and to synthesize the findings at a higher level of abstraction [73, 77].

The cross-case analysis followed Miles and Huberman’s ‘causal network approach’ and was based on the causal network models developed during the within-case analysis [73]. Patterns of the flow of variables were isolated from each case and then matched and compared across the cases through the use of a matrix which identified both comparability and variability between the cases. The quantitative data relating to utilization of acute care services were compared using one-way between-group analyses incorporating analysis of covariance (ANCOVA) [80, 81].

The qualitative data from the interviews were digitally recorded, transcribed verbatim, and then coded and thematically analysed. The constant comparative method [82] was used for coding, which compares incident to incident, incident to concept, and concept to concept to create higher level abstractions that described the emerging concepts, patterns, themes, and trends. Documents and qualitative data from the archival records were summarized for their context and significance.

The quantitative data from the practices reflected the changes in practice patient volume between the two time periods and the health authority data quantified emergency presentations and hospital admissions from emergency for the patients in each of these three practices. The health authority data included 10,063 emergency presentations and 1,461 hospital admissions; these were entered into SPSS version 17 for analysis.

Each case study site had a different practice volume for each of the two time periods, and in one case a different number of practitioners. Therefore, to allow for comparison, all data were standardized to the number of emergency visits or hospital admissions per 100 patients. The number of patient emergency visits was calculated on a monthly basis for each practitioner and then totalled for each time period. The number of hospital admissions from emergency was calculated on a monthly basis for each practitioner, and a total for the practice, for each time period. These data were then analysed to create descriptive statistics. The statistics created from the before and after data included mean number of emergency visits per month per practice, mean number of visits per month per practitioner, and the mean number of admissions per month per practice. These data were then used to calculate the percentage change between the before and after data. Bivariate analyses of before and after means at the case level were undertaken using paired sample *t*-tests. Depending on the number of practitioners at each site between 7 and 28 paired samples were included in the analysis for each site.

After the two types of data had been analysed independently the results were integrated to form meta-inferences within each case and ‘causal network’ models were created [73, 77]. These causal network models identified the variables in each individual case and the relationships among the variables. Cross-case analysis of the three cases was then undertaken to identify similarities and differences among the cases. The cross-case analysis followed the causal network analysis approach and was based on the causal networks developed for each case [73]. More detail on the causal network analysis will be described in a subsequent publication.

### Rigour

Although this research was based on both Yin’s [74] and Miles and Huberman’s [73] case study methodology, the use of Yin’s recommended criteria for assessing quality did not fit well with the types of data collected in this study. Instead, Teddlie and Tashakkori’s [77] integrated framework of inference quality was used, which incorporates both qualitative and quantitative standards to make a judgement about inference transferability. This integrated framework is based on two aspects of quality: design quality and interpretive rigour. The design quality was addressed through the use of multiple sources of evidence and the development of an auditable data trail. The interpretive rigour was addressed through the within-case analysis and then cross-case analysis which identified emerging patterns and a typical story of the changes identified since the enactment of the NP role in these practices. Inference transferability was addressed as the findings from this study were expected to be transferable to other settings in which the NP role had been established in a collaborative practice model with GPs.

## Results and discussion

Because this research was structured around a five-level ecological framework (NP role, other practitioners such as GPs and allied health professionals, practice organization, community, and health system), the results are presented in these levels. First, the individual NP roles were explored and described to provide context to the study. Then the NP’s actions and the resulting outcomes were identified at the practitioner, practice organization, community, and health authority levels. The following findings are from the cross-case analysis of the three cases which represents a synthesis across the cases.

### NP role and practice

Across all the cases, the NP role represented the full scope of family primary care practice, consistent with the scope of practice and standards expected by the provincial regulatory body. Four main activities were identified: primary care, educational activities, administrative/managerial activities, and research.

Primary care was the principal activity and accounted for 80% to 90% of NP time. This primary care involved both the general practice population and a population specific to the interests of the individual NP and/or the needs of the particular community. These specific populations included marginalized populations with mental health, HIV, and addiction concerns; seniors and frail elderly; patients with chronic diseases, particularly heart failure and diabetes; and women with reproductive health care needs and issues. In every case, there was also a community focus to the role. This involved either the delivery of primary care outside the office setting to populations that were previously not well served or community-based education, or both. These were primary care activities not typically provided by the GPs in the practices.

The NPs were able to spend more time with each patient visit and many changes were noted to be the result of this increased time. This additional time was described by other practitioners as “*bringing a new dimension to the provision of care*”. NP appointments were 20 to 30 minutes compared to the normal FFS visit of 10 minutes. This facilitated addressing more concerns in one visit, extra time being available for improved patient education, which generally focused on health promotion and illness prevention, and more time for in-depth examination of the patient’s situation or more comprehensive care. All of these were acknowledged by the other practitioners to lead to better care planning, reduced need for future appointments, improved patient engagement, and developed more of a partnership between the patient and the provider in the provision of their care. The NPs also demonstrated a different way of practising, which comprised enhanced skills in communication, more flexibility and openness to different lifestyles, and a willingness to take on more roles, including providing case management.

### Other practitioners

At the practitioner level, changes occurred in the other practitioners’ provision of care to patients, interprofessional communication, collaborative practice, and job satisfaction. The changes that were noted to have occurred included:

1. In every case, the presence of the NP allowed some of the other practitioners to modify their day to day activities, to focus on their preferred setting, and their personal interests and strengths.
2. In all cases, interprofessional communication improved and in most cases there was development of teamwork, shared care, and shared responsibility for care among the practitioners.
3. In the majority of cases, the style of practise changed from a siloed approach, in which the practitioners work side by side rather than together, to a group style of practise that involved a collaborative relationship in which the patients moved back and forth between the GPs and the NP.
4. The majority of other practitioners also reported improved job satisfaction. The main reasons given for this improved job satisfaction were “*easing the burden of trying to do everything yourself*” and “*working together to use each other’s strengths to get the work done*”. This improved job satisfaction was acknowledged to have had a positive impact on these practitioners’ desire to stay in their work environment.

### Practice organization

At the practice organizational level changes were identified in two key areas: patient access to the practice and workplace relationships, knowledge, and teamwork.

Patient access to the practices improved in three ways:

1. There was a decrease in wait times for appointments for existing patients. Before the implementation of the NP role, the wait time for an appointment ranged from one to six weeks across the three cases. After the implementation, every practice was able to offer same day appointments for urgent and many other patients, and all patients were able to get appointments within three days. This improved availability was directly related to NP role implementation and noted by office staff to have decreased the number of patients they referred to emergency.
2. There was increased access for patients to a primary care practice. Across the cases, the volume of patients increased by between 400 and 800 patients per GP (see Table 2 for specific details of practice volumes changes). These were either new patients to the practice or patients that were able to be retained by the practice even though they had lost their GP due to a reduction in the number of GPs working in the practice. The increase in practice volume was made possible by the introduction of the NP role to the practice.

**Table 2:** **Comparison of emergency presentations pre-/post-NP role implementation**

	Case 1	Case 2	Case 3
Practice volume 2007/2008	3,660	2,500	1,200
Total number emergency visits 7 months 2007/2008	1,880	1,387	451
Practice volume 2011	4,048	1,850	1,954
Total number emergency visits 7 months 2011	1,300	612	391
% Decrease in emergency visits between 2007/2008 and 2011	37.5%	40.4%	46.8%
*P* value (two-tailed) *P* <0.05	0.000	0.000	0.000
Eta squared	0.85	0.74	0.93

3. There was now a greater choice of provider and patients were actively choosing the practitioner they wanted to see on a particular visit.

In the area of improved workplace relationships, knowledge, and teamwork, both GPs and office staff acknowledged that the NPs had improved communication and teamwork with the support staff of the practice. Staff and physicians described the NPs as the “*bridge between the staff and the physicians*”. They also acknowledged that the staff members were more comfortable bringing their questions and concerns to the NP as a link to the physicians, and that the physicians were happy with this. The NPs were also credited with improving the knowledge of the office staff which allowed them to feel more engaged with the work of the practice and to feel that they were more of a team member.

### Community

At the community level, access to primary care improved for populations that were previously not well served through the traditional FFS office visit, in particular frail elderly and marginalized individuals who are harder to serve. Some of these patients had previously been obtaining their primary care through the use of emergency departments. Home and care facility visits for frail elderly were now generally attended to by the NPs. This new availability of primary care services in homes and other community locations was appreciated by patients, families, community nurses, and other community teams and services.

Drop-in clinics were also implemented associated with community health events or involved with other community partners. These clinics captured populations that had not previously been accessing these services and generally were focused on health education and preventative and screening services.

The NPs were also credited with developing links between the practice and the community. Staff commented that, prior to the arrival of the NPs, none of the practices had much awareness or involvement with what was happening in their community. This changed, and in the majority of cases the NP became the link between the practice and the community, resulting in a greater understanding and sense of connection between the practice and community services.

### Acute care services

At the health authority level, two types of impacts were investigated: emergency presentations and admissions to hospital from emergency.

One of the key features of this research was the analysis of the health authority data relating to changes in the utilization of local acute care services by the patients of these practices following introduction of the NP role. Across the practices, there was a marked decrease in the number of emergency presentations when the pre and post NP role implementation data were compared. These decreases were statistically significant and ranged from 37% to 47%. Table 2 provides a comparison of the emergency presentations pre- and post-NP role implementation across the three cases.

There was also a marked decrease in hospital admissions from emergency after the implementation of the NP role. The decreases ranged from 74% to 88% and were statistically significant for all cases. Table 3 provides a cross-case comparison of the hospital admissions from emergency presentations pre- and post-NP role implementation.

**Table 3:** **Comparison of hospital admissions from emergency presentations pre-/post-NP role implementation**

	Case 1	Case 2	Case 3
Total number admissions 7 months 2007/2008	65	50	31
Total number admissions 7 months 2011	19	20	6
% Decrease in total admissions between 2007/2008 and 2011	73.6%	80.0%	88.0%
*P* value (two-tailed) *P* <0.05	0.000	0.000	0.000
Eta squared	0.48	0.49	0.91

Despite these findings, it is important to acknowledge that other confounding factors may have also have influenced these decreases in acute care utilization. However, the conclusion that the NP’s role contributed to this outcome is strengthened by the triangulation of the qualitative and quantitative findings, the fact that three distinct cases produced the same results, and, in two cases, the practice volume increased while the total number of acute care presentations decreased.

### Summary of key findings

**Figure 1:**
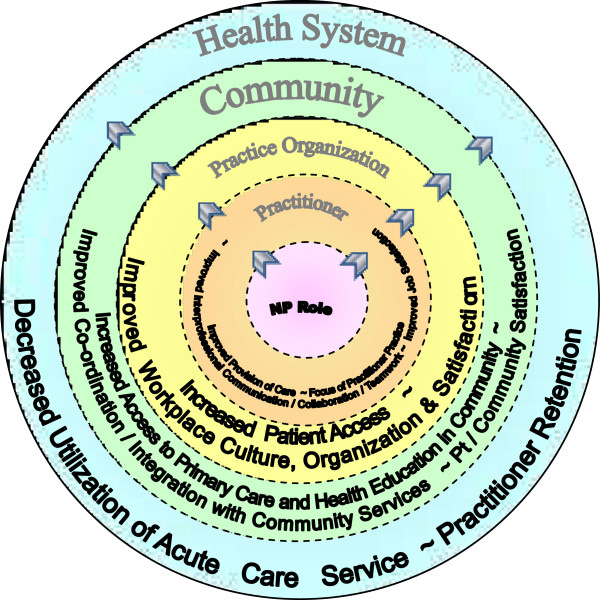
**Framework depicting key findings.**

The key findings from each level of the ecological framework are depicted in Figure 1. In this figure, the lines between the levels are dotted reflecting the interconnected relationships that were found to exist among the outcomes at different levels and that outcomes at one level created outcomes at another level. The arrows indicate that the predominant direction of movement of influences was from the NP role in the centre of the framework to the health system level at the perimeter of the nested layers of relationships.

## Discussion

This study confirmed that previous findings regarding the positive impact of NPs in other primary care settings were also demonstrated in the FFS setting. These included more time spent with patients [83–85], more comprehensive and holistic care [86], increased patient education and teaching [87, 88], increased knowledge and use of community resources [18], increased patient engagement and partnership [89], and, in some cases, improvements in chronic disease management [44, 90]. This study also confirmed the continuation of the findings from the 12-month post-implementation qualitative local program evaluation of two of these case sites (not part of this study) which found increased patient access to care and provider satisfaction [91].

New knowledge was also identified from these FFS settings. This included that the NP role was more focused on direct patient care than has been found in other primary care settings [16–18, 92] and as a result less time was available to be devoted to the other dimensions of the role that are defined as essential in advanced nursing practice [93] such as research. However, a larger study would be needed to determine whether this finding is consistent across other practices. This has implications for how the other expected components of the advanced practice NP role are able to be met in this setting, and whether the expectations for activities such as research are feasible and how they could be accomplished. More exploration of this issue is needed.

Secondly, this study exemplified the diversity of health care needs that can be met by a salaried NP role in a FFS practice. Each role had at least two foci – the general population and an additional focus specific to the interests or expertise of the NP and the needs of the community. This allowed more services to be provided to ‘harder-to-serve populations’, such as the frail elderly and those with mental health concerns, addictions, and HIV. This finding may be particularly important in the rural and remote setting where the size of these populations is unlikely to be large enough to justify the cost of providing specific services. It may also provide a means whereby home visits to frail and homebound elderly can be provided through their traditional primary care practices.

This study highlighted how the extra time available for each patient with this type of NP role can enhance patient care in the FFS model. FFS is unlikely to change in this province as the predominant funding mechanism for primary care practice in the foreseeable future, therefore, the benefits this additional time can bring to this model of care is an important contribution. However, it is important to note the combined FFS/salary model has financial implications that have not been fully investigated to determine whether it is the best model, or whether there are alternative models that can be equally, or more, cost effective.

The NP role also facilitated the development of shared care and shared responsibility for care between the practitioners. One of the key factors to achieving shared care was the high level of trust and respect shown by their physician colleagues for the NPs’ clinical competence. Shared care also facilitated the move from a siloed to a group style of practice. This move has been found in previous research to be important for the development of a more complete model of primary care incorporating health promotion, prevention, continuous, and more comprehensive care [94]. This change has been acknowledged to be a challenging process [95]. The NP’s role in facilitating this change has not been previously identified.

The NP role also resulted in improvements in workplace culture, organization, and job satisfaction for the practice staff. These were important outcomes for organizational effectiveness and successful service provision.

This study further supported some of the benefits at the community level that the NP role has demonstrated [16–18, 92, 96]. These included the provision of primary care to those most at risk and in need of multiple services, and the ability to link primary care practices with community services.

A number of the findings from this study appeared to be related to the methods of remuneration used for the different practitioners. The salaried nature of the NP role within this setting allowed the NP to address the needs of the harder-to-serve population that are not well served within the FFS model, to have a community focus that would otherwise not have been possible, and to provide the extra time for patient visits that had associated benefits. The mixing of these two models appears to bring significant benefits that might not otherwise be achieved.

The extent of potential increase in access to primary care and decrease in acute care utilization was quantified in this study. While the NP’s role in achieving this outcome cannot be confirmed definitively in this type of design, the triangulation of the qualitative and quantitative findings across these three cases strengthens the evidence of the value that the NP role can potentially bring to reductions in acute care utilization.

### Limitations

There were limitations associated with this study. Firstly, there were only a limited number of case sites that were undertaking this collaborative practice model of care; this resulted in only a small sample being available to include in the study and on which to draw conclusions about the NP role in FFS practice. Secondly, no patients participated in the qualitative data collection; therefore, claims about improvements in patient outcomes and satisfaction that were reported by others cannot be validated.

Because of the limited information about outcomes associated with the NP role in FFS GP clinics, and the need to provide context to the NP role, a case study design was chosen. The qualitative nature of this design does not lend itself to confirming that action X caused result Y; nevertheless, this methodology has allowed knowledge to be developed about how and why some events and situations affect others and identified the mechanisms that establish what actions preceded what events. This type of pattern matching is acknowledged as providing support for making justified inferences about the causal direction of relationships between and among variables and concepts using qualitative data [74, 97]. The future use of an experimental design may be used to verify with greater certainty a direct causal relationship between the NP role and these outcomes.

This study did not explore the economics associated with this collaborative practice model, and as noted earlier, there is currently little information available in this area. As a result, economic impacts cannot be used as a driver for changes in practice and policy directions. This is an area where further research needs to be undertaken.

## Conclusions

This study has shown that the addition of a NP to the predominant delivery model for primary care services, namely FFS, can improve available care and result in substantial positive effects on fundamental problems facing the health system. Careful mapping of the relationships among variables and concepts using mixed methods analysis in a case study design demonstrated positive outcomes of NP practice at all levels of the system, both within and externally, to these primary care practices. The provision of care for patients was reported to have improved; relationships and teamwork involving the practitioners and the practice were perceived as enhanced; access to the practice for patients was documented to have significantly increased; the practices were perceived by many to become more connected with their communities; and the utilization of acute care services significantly decreased for the patients of the practices. These outcomes associated with the NP/GP collaborative practice primary care model demonstrate the importance of the NP role in this setting and support a conclusion that it can play an important role in meeting the primary care needs of the population.

## Authors’ information

AR is a contracted researcher with the School of Health and Human Sciences, Southern Cross University, Lismore, Australia. At the time of this research AR was a doctoral candidate in the School of Nursing, University of Victoria, British Columbia, Canada. MM is a Professor in the School of Nursing, University of Victoria, British Columbia, Canada.

## Abbreviations

FFS: Fee for service
GP: General practitioner
NP: Nurse practitioner.
